# Supplementary material for: Metabolic Potential of Microbial Communities in the Hypersaline Sediments of the Bonneville Salt Flats
Source: mSystems. 2022 Nov 15;7(6):e00846-22. doi: 10.1128/msystems.00846-22 (PMC9765009; doi:10.1128/msystems.00846-22)
Supplement: FIG S2 [file msystems.00846-22-s0001.pdf]

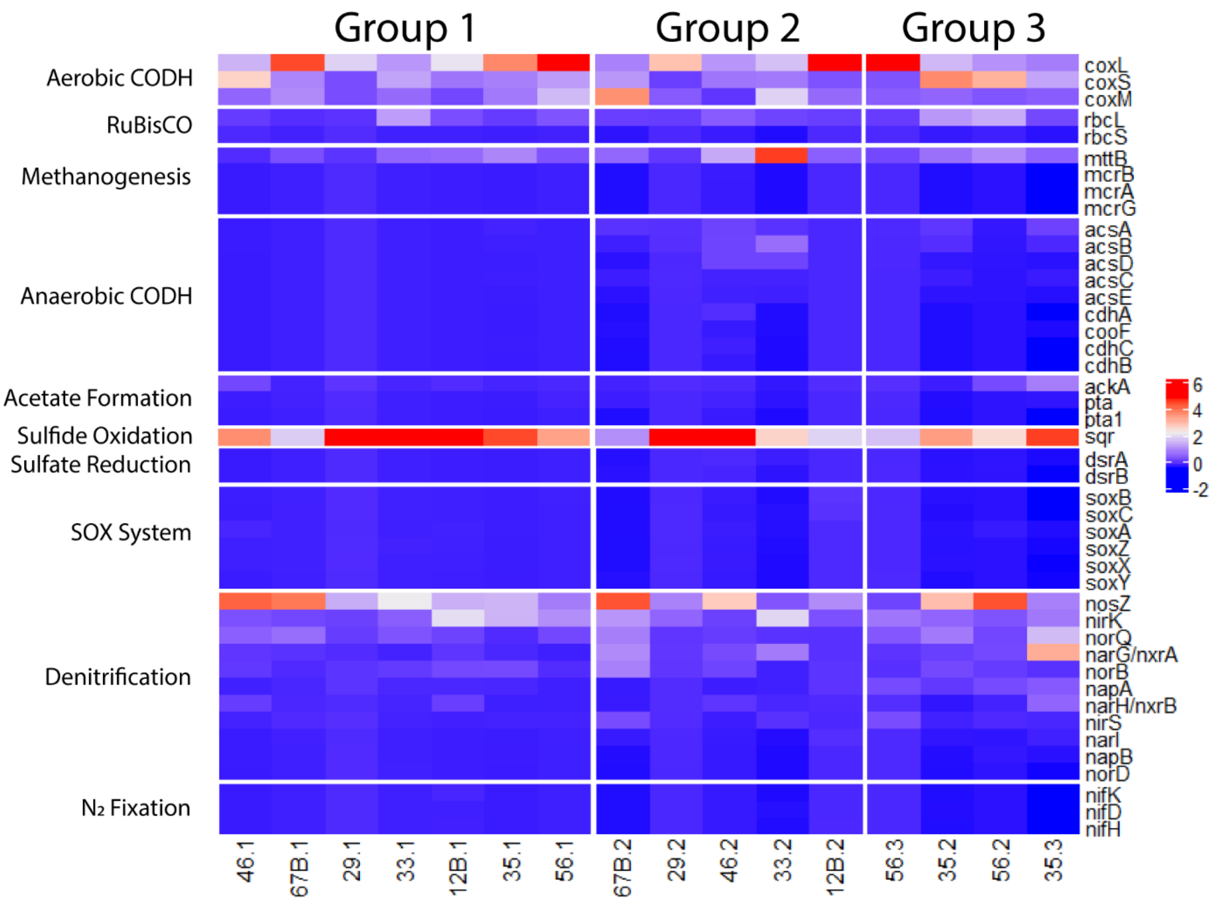

Figure S2 Heatmap of normalized gene abundance (as scaled *TPM* values) for genes and complexes of interest at the BSF. Names on the left represent KEGG pathways associated with these genes, but overlaps with other functions are possible.
