# Supplementary material for: Metabolic Potential of Microbial Communities in the Hypersaline Sediments of the Bonneville Salt Flats
Source: mSystems. 2022 Nov 15;7(6):e00846-22. doi: 10.1128/msystems.00846-22 (PMC9765009; doi:10.1128/msystems.00846-22)
Supplement: FIG S3 [file msystems.00846-22-s0003.pdf]

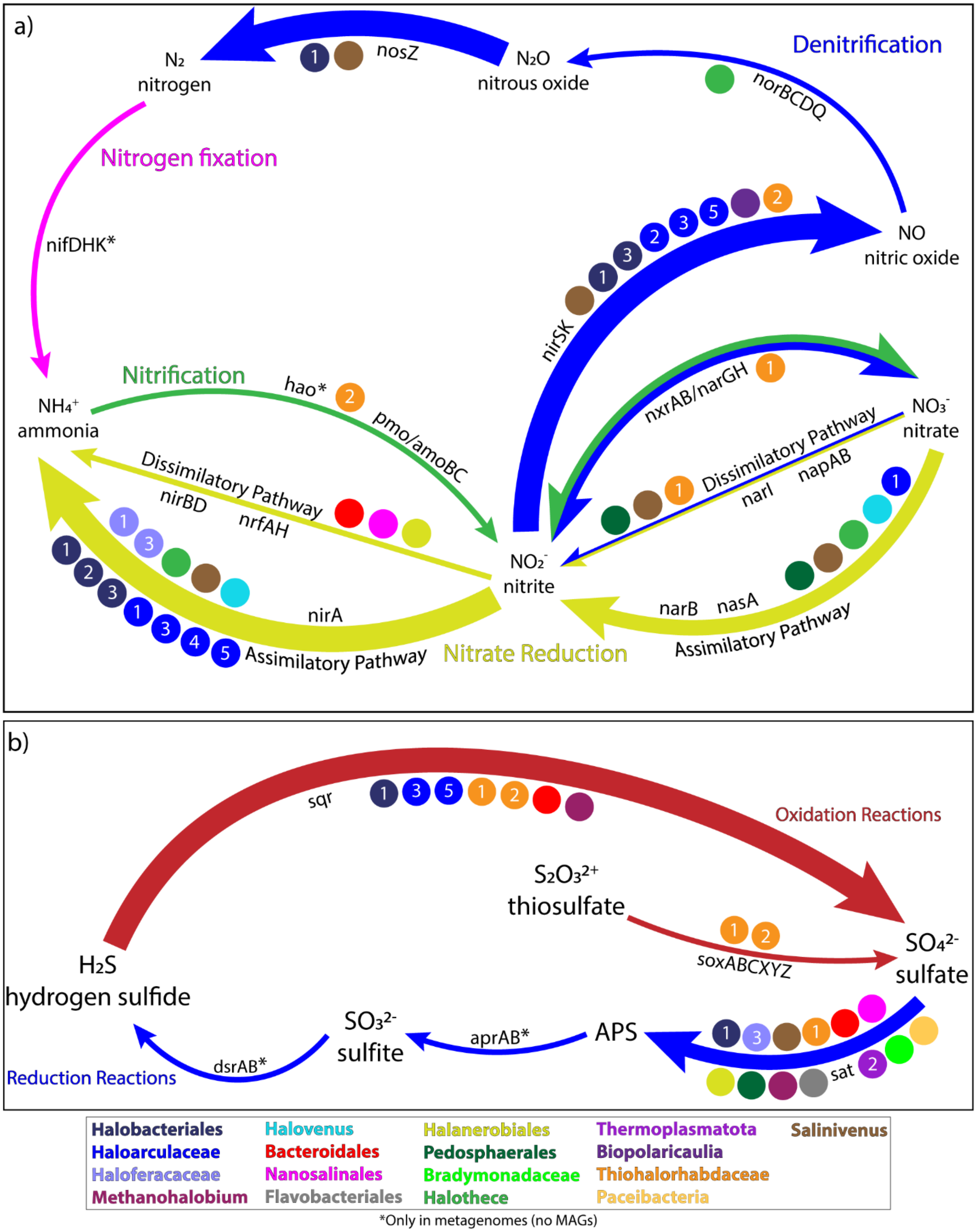

Figure S3 a) Overview of the complete BSF nitrogen cycle and associated genes and species. Colored circles represent MAGs containing at least 1 gene in the indicated pathway. Arrows are colored by

process. The size of the arrow corresponds to the gene abundance in the sample metagenomes, where larger arrows indicate more abundant enzymes responsible for that reaction in the nitrogen cycle. b) Overview of the complete BSF sulfur cycle and associated genes and species. Colored circles represent MAGs containing at least 1 gene in the indicated pathway. Arrows are colored by process. The size of the arrow corresponds to the gene abundance in the sample metagenomes, where larger arrows indicate more abundant enzymes responsible for that reaction in the sulfur cycle.
