# Supplementary material for: Metabolic Potential of Microbial Communities in the Hypersaline Sediments of the Bonneville Salt Flats
Source: mSystems. 2022 Nov 15;7(6):e00846-22. doi: 10.1128/msystems.00846-22 (PMC9765009; doi:10.1128/msystems.00846-22)
Supplement: FIG S4 [file msystems.00846-22-s0005.pdf]

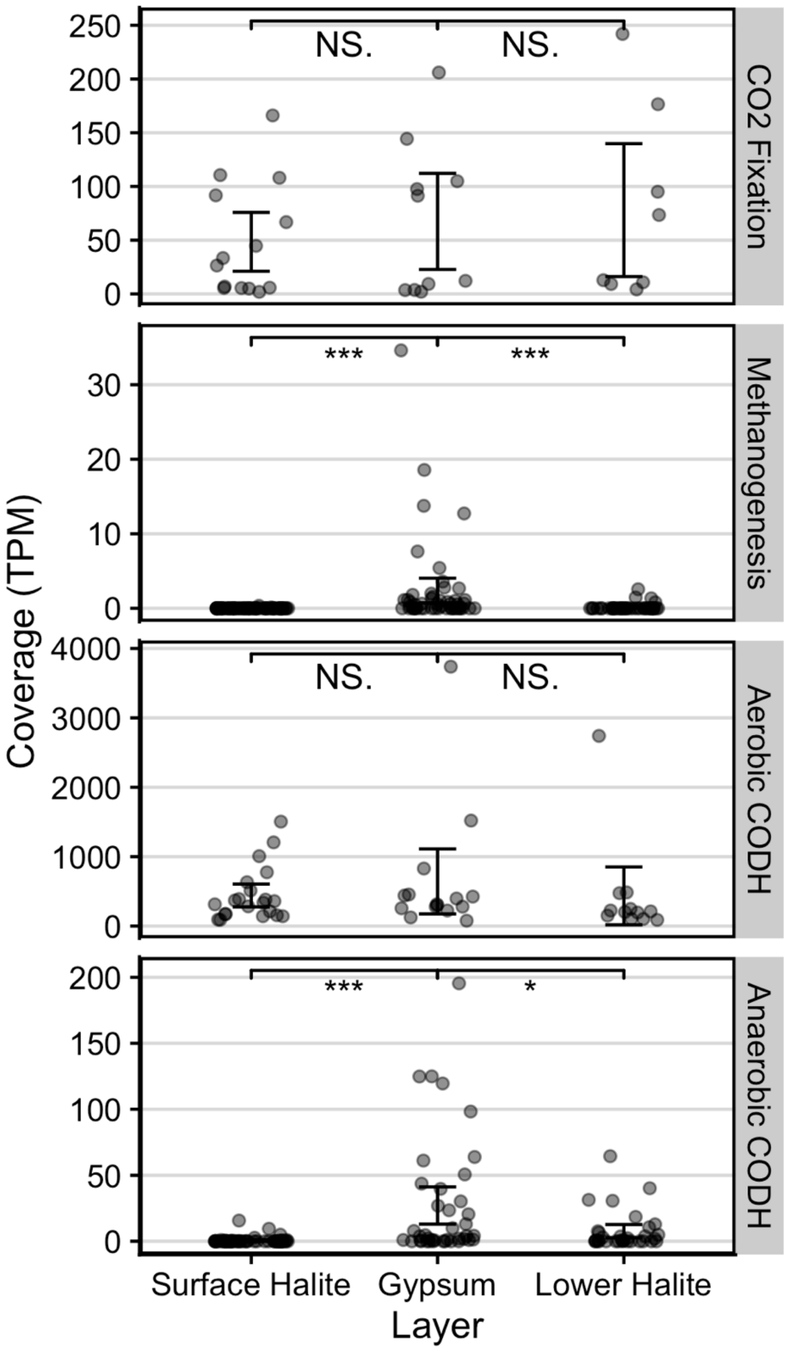

Figure S4A. BSF sediment layers (surface halite, gypsum, and lower halite) differ in their coverages of some, but not all, genes associated with **carbon metabolism**. Each data point represents the coverage of one predicted gene in one sample, with genes grouped by metabolic category and samples grouped by sediment layer. This plot is a summary of the same data reported in **Figure 2**. Significant differences between layers were tested with the Wilcoxon Rank Sum Test and are shown with asterisks (* = $p < 0.05$, ** = $p < 0.01$, *** = $p < 0.001$). Significant differences between Surface Halite and Lower Halite layers are not shown.

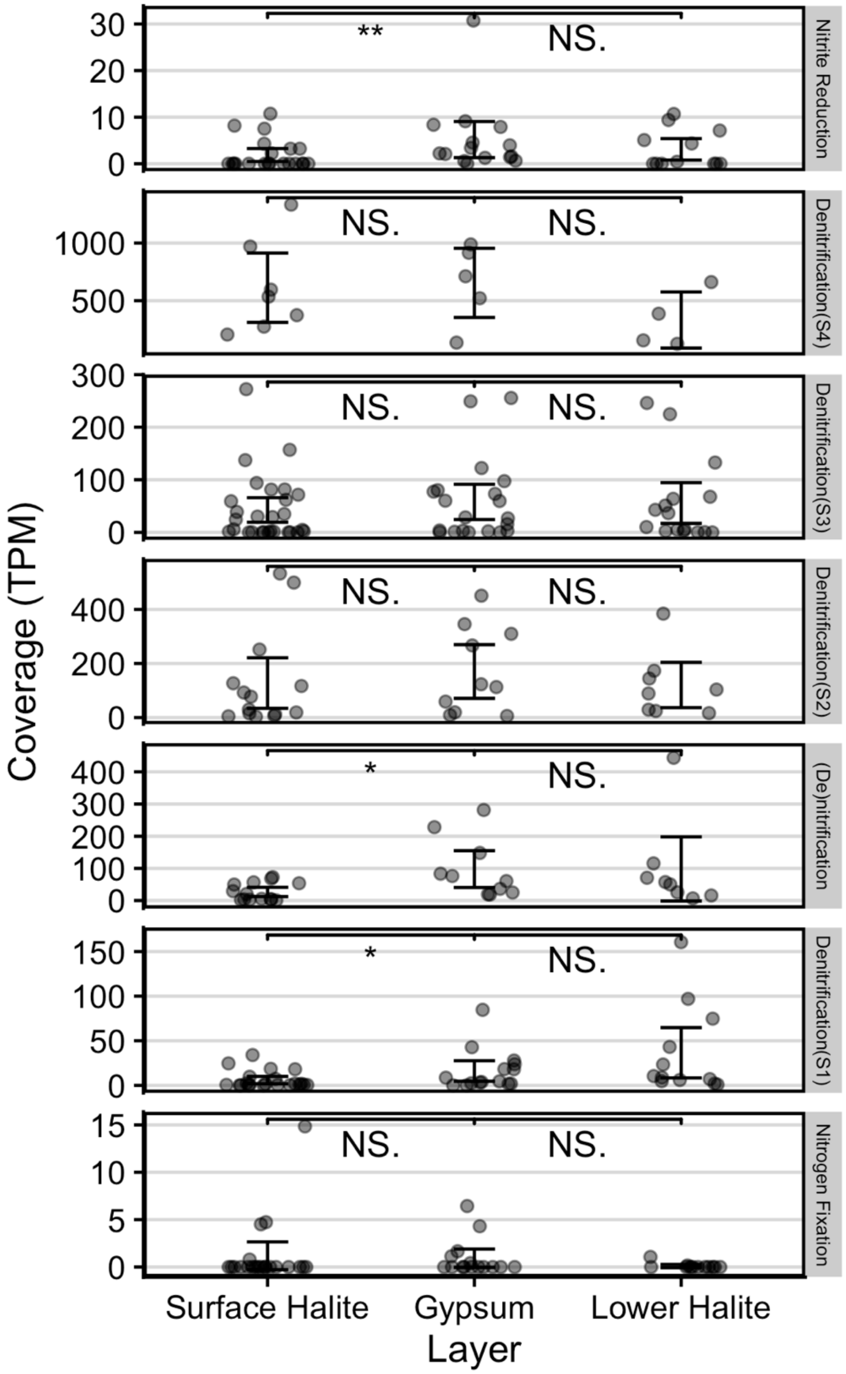

Figure S4B. BSF sediment layers (surface halite, gypsum, and lower halite) differ in their coverages of some, but not all, genes associated with **nitrogen metabolism**. Each data point represents the coverage of one predicted gene in one sample, with genes grouped by metabolic category and samples grouped by sediment layer. This plot is a summary of the same data reported in **Figure 4**. Significant differences between layers were tested with the Wilcoxon Rank Sum Test and are shown with asterisks (* = $p < 0.05$, ** = $p < 0.01$, *** = $p < 0.001$). Significant differences between Surface Halite and Lower Halite layers are not shown.

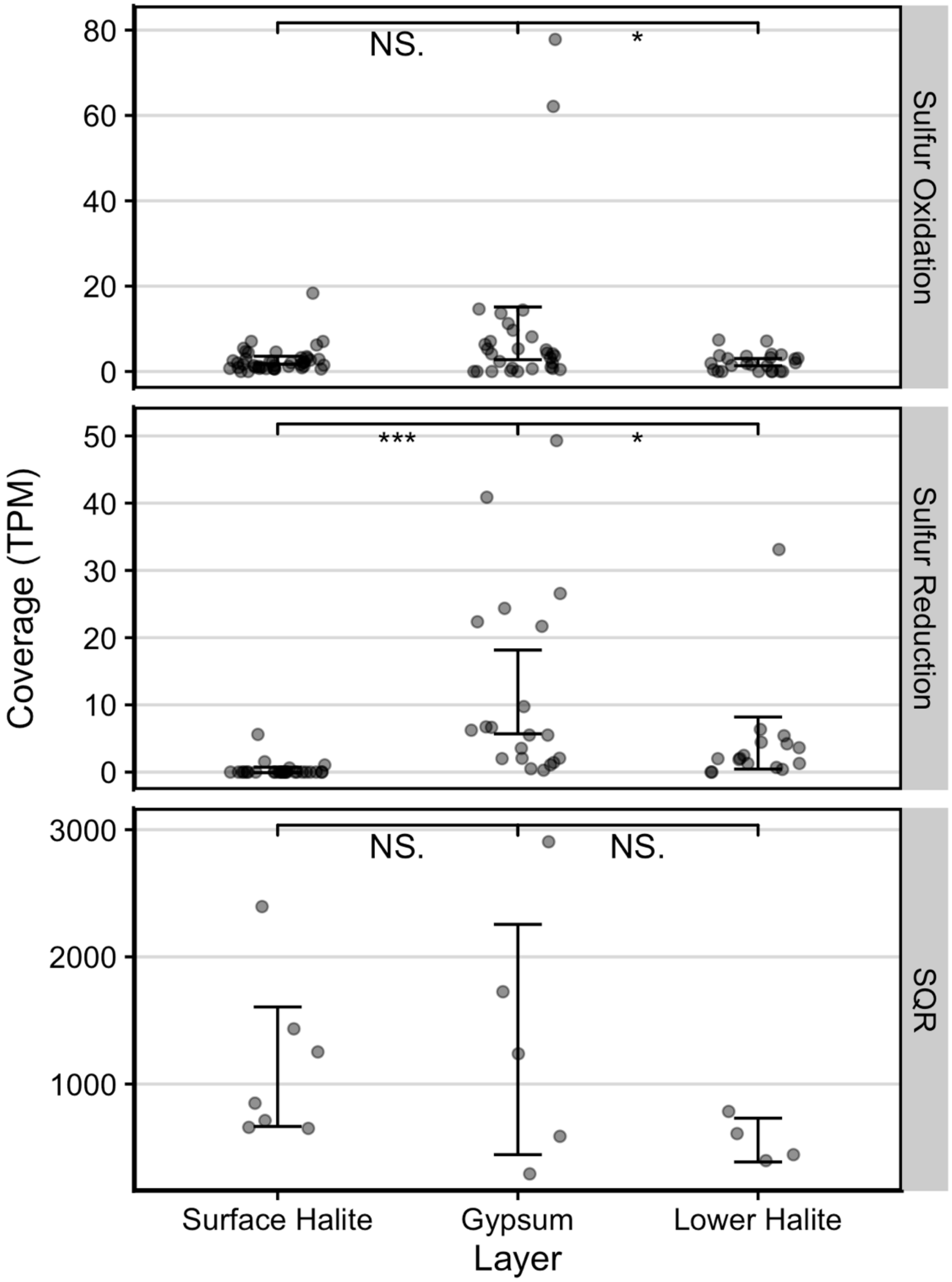

Figure S4C. BSF sediment layers (surface halite, gypsum, and lower halite) differ in their coverages of some, but not all, genes associated with **sulfur metabolism**. Each data point represents the coverage of one predicted gene in one sample, with genes grouped by metabolic category and samples grouped by sediment layer. This plot is a summary of the same data reported in **Figure 5**. Significant differences between layers were tested with the Wilcoxon Rank Sum Test and are shown with asterisks (* = $p < 0.05$, ** = $p < 0.01$, *** = $p < 0.001$). Significant differences between Surface Halite and Lower Halite layers are not shown.
