# Supplementary material for: Metabolic Potential of Microbial Communities in the Hypersaline Sediments of the Bonneville Salt Flats
Source: mSystems. 2022 Nov 15;7(6):e00846-22. doi: 10.1128/msystems.00846-22 (PMC9765009; doi:10.1128/msystems.00846-22)
Supplement: FIG S1 [file msystems.00846-22-s0002.pdf]

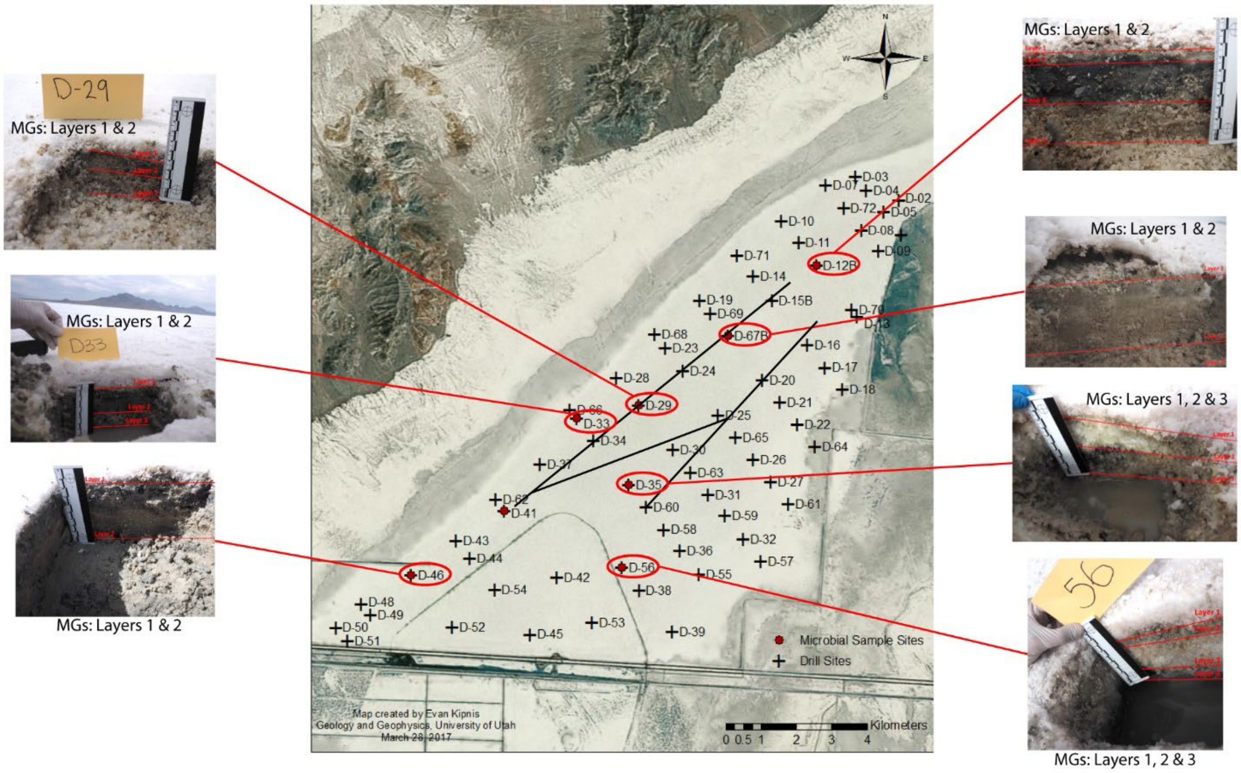

Figure S1 Sampling locations and field photos of sampling sites. Black lines indicate approximate location of the racetrack at the time of sampling. The sediment layers at each site that were included in the metagenomic study are labeled.
